# (2*E*)-3-(3-Bromo-4-meth­oxy­phen­yl)-1-(4-fluoro­phen­yl)prop-2-en-1-one

**DOI:** 10.1107/S1600536811012505

**Published:** 2011-04-13

**Authors:** Grzegorz Dutkiewicz, B. P. Siddaraju, H. S. Yathirajan, B. Narayana, Maciej Kubicki

**Affiliations:** a Department of Chemistry, Adam Mickiewicz University, Grunwaldzka 6, 60-780 Poznań, Poland; b Department of Studies in Chemistry, University of Mysore, Manasagangotri, Mysore 570 006, India; c Department of Studies in Chemistry, Mangalore University, Mangalagangotri 574 199, India

## Abstract

In the title compound, C_16_H_12_BrFO_2_, the dihedral angle between the aromatic rings is 23.75 (12)° and the dihedral angle between the prop-2-en-1-one fragment and the fluorobenzene ring is 20.9 (2)°. In the crystal, only van der Waals interactions occur.

## Related literature

For the normal probability plot test, see: Abrahams & Keve (1971 ▶); Cromer (1974 ▶). For the influence of the substituents on the geometry of the phenyl ring, see: Domenicano & Murray-Rust (1979 ▶); Domenicano (1988 ▶). For a closely related structure, see: Dutkiewicz *et al.* (2011 ▶).
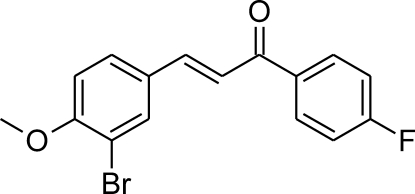


## Experimental

### 

#### Crystal data

C_16_H_12_BrFO_2_
*M* *_r_* = 335.17
Monoclinic, 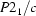

*a* = 11.056 (2) Å
*b* = 4.1110 (15) Å
*c* = 30.825 (5) Å
β = 96.76 (2)°
*V* = 1391.3 (6) Å^3^
*Z* = 4
Mo *K*α radiation
μ = 2.96 mm^−1^
*T* = 295 K
0.5 × 0.4 × 0.15 mm

#### Data collection

Agilent Xcalibur Eos diffractometer
Absorption correction: multi-scan (*CrysAlis PRO*; Agilent, 2010 ▶) *T*_min_ = 0.507, *T*_max_ = 1.000
6983 measured reflections
2887 independent reflections
1918 reflections with *I* > 2σ(*I*)
*R*_int_ = 0.031

#### Refinement

*R*[*F* ^2^ > 2σ(*F* ^2^)] = 0.044
*wR*(*F* ^2^) = 0.092
*S* = 1.02
2887 reflections
182 parameters
H-atom parameters constrained
Δρ_max_ = 0.32 e Å^−3^
Δρ_min_ = −0.52 e Å^−3^

### 

Data collection: *CrysAlis PRO* (Agilent, 2010 ▶); cell refinement: *CrysAlis PRO*; data reduction: *CrysAlis PRO*; program(s) used to solve structure: *SIR92* (Altomare *et al.*, 1993 ▶); program(s) used to refine structure: *SHELXL97* (Sheldrick, 2008 ▶); molecular graphics: *SHELXTL* (Sheldrick, 2008 ▶); software used to prepare material for publication: *SHELXL97*.

## Supplementary Material

Crystal structure: contains datablocks I, global. DOI: 10.1107/S1600536811012505/dn2670sup1.cif

Structure factors: contains datablocks I. DOI: 10.1107/S1600536811012505/dn2670Isup2.hkl

Additional supplementary materials:  crystallographic information; 3D view; checkCIF report

## Figures and Tables

**Fig. 1.**
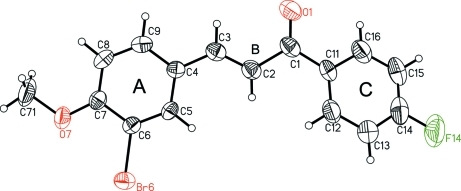
Anisotropic ellipsoid representation of the compound I together with atom labeling scheme. The ellipsoids are drawn at 50% probability level, hydrogen atoms are shown as spheres of arbitrary radii.

**Fig. 2.**
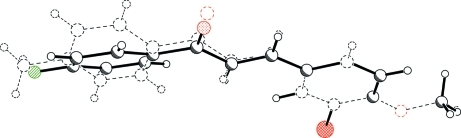
The comparison of the molecules of I (solid) and its 4-methoxy analogue (Dutkiewicz et al., 2011; solid); the central prop-1-ene-3-one fragments were fitted onto one another (SHELXTL (Sheldrick, 2008)).

## supplementary crystallographic information

### Comment

As a part of our ongoing studies on chalcone derivatives (*e.g.* Dutkiewicz *et al.*, 2011) we have synthesized (2*E*)-3-(3-Bromo-4-methoxyphenyl)-1-(4-fluorophenyl)prop-2-en-1-one (I, Scheme 1).

The geometry of the molecule of I is very similar to that of previously reported (2*E*)-3-(3-Bromo-4-methoxyphenyl)-1-(4-methoxyphenyl)prop-2-en-1-one (Dutkiewicz *et al.*, 2011). The bond lengths and angles in both compounds are very similar; a majority of them differ by less than 3σ, and even the results of the normal probability plot test (Abrahams & Keve, 1971; International Tables for X-ray Crystallography, vol. IV (Cromer, 1974) confirm that the differences between the molecules are mainly of statistic nature. The correlation coefficient *R*^2^ between the set of experimental differences between the geometrical parameters and the theoretical values for pure statistical distribution is 0.97 for the bond lengths (excluding C14—C141 and C14—F14 bonds, of course) and 0.94 - for angles. From the normal probability plot for bond angles it is obvious that the largest, and certainly not random, differences are observed within the phenyl ring with different substituents. This is consistent with the old observation of Domenicano and Murray-Rust (1979) that substituents to the phenyl ring influence much more intraannular bond angles than the bond lengths. The nature of substituents causes completely different bond angles pattern within the phenyl ring, in agreement with the values given by Domenicano (1988) which highlight quite opposite natures of methyl and fluorine groups.

More significant differences are observed at the level of torsion angles, that means that the overall conformations of both compounds differ. The shape of these molecules can be described by the dihedral angles between three planar fragments (*cf.* Fig. 1) 1-bromo-2-methoxyphenyl ring (A), the central prop-2-en-1-one chain (B), and the fluoro-phenyl ring (C). In both cases the dihedral angles between A and B planes are comparable, and in both cases the bridging chain is not significantly tilted out of the plane of the A ring, the orientations of rings C are really different: it is almost coplanar with the B - bridge for methyl derivative while it makes with the plane of prop-2-en-1-one group the significant dihedral angle of 20.9 (2)° in I. The comparison of both molecules, fitted onto the central C1O1C2C3 plane, is shown in Fig. 2.

In the structure of I, contrary to 1-(4-methoxyphenyl) derivative, where we observed quite a rich structure of weak interactions, there are virtually no specific interactions which might play a role in the designing of the crystal structure. Therefore only close packing requirements and van der Waals forces are involved in the crystal structure.

### Experimental

3-Bromo-4-methoxybenzaldehyde (2.15 g, 0.01 mol) was mixed with 1-(4-fluorophenyl)ethanone (1.38 g, 0.01 mol) and dissolved in ethanol (40 ml). To the solution, 4 ml of KOH (50%) was added. The reaction mixture was stirred for 6–10 h. The resulting crude solid was filtered, washed successively with distilled water and finally recrystallized from ethanol (95%) to give the pure chalcones. Crystals suitable for *x*-ray diffraction studies were grown by the slow evaporation from acetone solution (mp.: 140°C). Composition: Found (Calculated): C_16_H_12_BrFO_2_:*C*: 57.26 (57.34); H: 3.57 (3.61)

### Refinement

The hydrogen were placed geometrically, in idealized positions, and refined as rigid groups with their *U*_iso_(H) = 1.2*U*_eq_(C) with distances C—H = 0.93Å of the appropriate carrier atom (*U*_iso_(H) = 1.5*U*_eq_(C) with distances C—H = 0.96Å for methyl H).

### Crystal data

**Table:**

C_16_H_12_BrFO_2_	*F*(000) = 672
*M**_r_* = 335.17	*D*_x_ = 1.600 Mg m^−^^3^
Monoclinic, *P*2_1_/*c*	Mo *K*α radiation, λ = 0.71073 Å
Hall symbol: -P 2ybc	Cell parameters from 2417 reflections
*a* = 11.056 (2) Å	θ = 3.1–28.0°
*b* = 4.1110 (15) Å	µ = 2.96 mm^−^^1^
*c* = 30.825 (5) Å	*T* = 295 K
β = 96.76 (2)°	Prism, colourless
*V* = 1391.3 (6) Å^3^	0.5 × 0.4 × 0.15 mm
*Z* = 4	

### Data collection

**Table:**

Agilent Xcalibur Eos diffractometer	2887 independent reflections
Radiation source: Enhance (Mo) X-ray Source	1918 reflections with *I* > 2σ(*I*)
graphite	*R*_int_ = 0.031
Detector resolution: 16.1544 pixels mm^-1^	θ_max_ = 28.0°, θ_min_ = 3.1°
ω scans	*h* = −13→14
Absorption correction: multi-scan (*CrysAlis PRO*; Agilent, 2010)	*k* = −5→5
*T*_min_ = 0.507, *T*_max_ = 1.000	*l* = −38→39
6983 measured reflections	

### Refinement

**Table:**

Refinement on *F*^2^	Primary atom site location: structure-invariant direct methods
Least-squares matrix: full	Secondary atom site location: difference Fourier map
*R*[*F*^2^ > 2σ(*F*^2^)] = 0.044	Hydrogen site location: inferred from neighbouring sites
*wR*(*F*^2^) = 0.092	H-atom parameters constrained
*S* = 1.02	*w* = 1/[σ^2^(*F*_o_^2^) + (0.045*P*)^2^] where *P* = (*F*_o_^2^ + 2*F*_c_^2^)/3
2887 reflections	(Δ/σ)_max_ = 0.001
182 parameters	Δρ_max_ = 0.32 e Å^−^^3^
0 restraints	Δρ_min_ = −0.52 e Å^−^^3^

### Special details

**Table:**

Geometry. All s.u.'s (except the s.u. in the dihedral angle between two l.s. planes) are estimated using the full covariance matrix. The cell s.u.'s are taken into account individually in the estimation of s.u.'s in distances, angles and torsion angles; correlations between s.u.'s in cell parameters are only used when they are defined by crystal symmetry. An approximate (isotropic) treatment of cell s.u.'s is used for estimating s.u.'s involving l.s. planes.
Refinement. Refinement of *F*^2^ against ALL reflections. The weighted *R*-factor *wR* and goodness of fit *S* are based on *F*^2^, conventional *R*-factors *R* are based on *F*, with *F* set to zero for negative *F*^2^. The threshold expression of *F*^2^ > 2σ(*F*^2^) is used only for calculating *R*-factors(gt) *etc*. and is not relevant to the choice of reflections for refinement. *R*-factors based on *F*^2^ are statistically about twice as large as those based on *F*, and *R*- factors based on ALL data will be even larger.

### Fractional atomic coordinates and isotropic or equivalent isotropic displacement parameters (Å^2^)

**Table:**

	*x*	*y*	*z*	*U*_iso_*/*U*_eq_	
C1	0.1287 (3)	0.1073 (8)	0.61784 (11)	0.0453 (9)	
O1	0.0290 (2)	0.2293 (7)	0.60968 (8)	0.0649 (7)	
C2	0.1926 (3)	0.1062 (8)	0.66297 (11)	0.0427 (8)	
H2	0.2639	−0.0139	0.6689	0.051*	
C3	0.1509 (3)	0.2716 (8)	0.69512 (11)	0.0397 (8)	
H3	0.0814	0.3956	0.6873	0.048*	
C4	0.1999 (3)	0.2836 (7)	0.74082 (10)	0.0328 (7)	
C5	0.3100 (3)	0.1388 (7)	0.75684 (10)	0.0340 (7)	
H5	0.3557	0.0294	0.7379	0.041*	
C6	0.3511 (3)	0.1577 (7)	0.80038 (11)	0.0342 (7)	
Br6	0.50370 (3)	−0.02669 (8)	0.820926 (12)	0.05081 (15)	
C7	0.2842 (3)	0.3147 (7)	0.83016 (10)	0.0370 (8)	
O7	0.3326 (2)	0.3123 (6)	0.87212 (7)	0.0539 (7)	
C71	0.2665 (4)	0.4821 (10)	0.90272 (13)	0.0834 (15)	
H71A	0.1882	0.3821	0.9033	0.125*	
H71B	0.2558	0.7053	0.8939	0.125*	
H71C	0.3114	0.4722	0.9313	0.125*	
C8	0.1740 (3)	0.4534 (7)	0.81422 (12)	0.0434 (8)	
H8	0.1263	0.5534	0.8333	0.052*	
C9	0.1346 (3)	0.4439 (7)	0.77032 (12)	0.0414 (8)	
H9	0.0622	0.5477	0.7600	0.050*	
C11	0.1903 (3)	−0.0519 (8)	0.58326 (10)	0.0414 (8)	
C12	0.3150 (3)	−0.1039 (9)	0.58687 (12)	0.0549 (10)	
H12	0.3634	−0.0397	0.6121	0.066*	
C13	0.3682 (4)	−0.2492 (10)	0.55362 (13)	0.0653 (11)	
H13	0.4519	−0.2840	0.5562	0.078*	
C14	0.2958 (4)	−0.3405 (10)	0.51699 (13)	0.0647 (11)	
F14	0.3475 (3)	−0.4875 (6)	0.48435 (9)	0.1010 (9)	
C15	0.1731 (4)	−0.2938 (10)	0.51172 (13)	0.0664 (11)	
H15	0.1257	−0.3590	0.4863	0.080*	
C16	0.1211 (3)	−0.1476 (10)	0.54502 (12)	0.0579 (10)	
H16	0.0375	−0.1121	0.5418	0.069*	

### Atomic displacement parameters (Å^2^)

**Table:**

	*U*^11^	*U*^22^	*U*^33^	*U*^12^	*U*^13^	*U*^23^
C1	0.044 (2)	0.050 (2)	0.040 (2)	−0.0048 (18)	−0.0020 (16)	0.0113 (17)
O1	0.0470 (15)	0.096 (2)	0.0495 (17)	0.0107 (15)	−0.0012 (12)	0.0108 (15)
C2	0.040 (2)	0.0474 (19)	0.040 (2)	−0.0022 (16)	0.0025 (16)	0.0054 (17)
C3	0.0342 (18)	0.0413 (19)	0.043 (2)	−0.0046 (15)	0.0030 (15)	0.0054 (17)
C4	0.0280 (16)	0.0331 (17)	0.0376 (19)	−0.0056 (14)	0.0059 (14)	0.0018 (15)
C5	0.0352 (18)	0.0330 (16)	0.0360 (19)	−0.0028 (15)	0.0135 (14)	−0.0030 (15)
C6	0.0311 (17)	0.0309 (16)	0.041 (2)	−0.0039 (14)	0.0074 (14)	−0.0027 (15)
Br6	0.0406 (2)	0.0546 (2)	0.0557 (2)	0.01012 (18)	−0.00083 (15)	−0.00365 (19)
C7	0.0423 (19)	0.0339 (17)	0.036 (2)	0.0000 (16)	0.0111 (15)	0.0006 (15)
O7	0.0669 (17)	0.0631 (16)	0.0318 (15)	0.0137 (13)	0.0064 (12)	−0.0071 (12)
C71	0.125 (4)	0.090 (3)	0.037 (2)	0.046 (3)	0.014 (2)	−0.013 (2)
C8	0.0422 (19)	0.041 (2)	0.050 (2)	0.0063 (17)	0.0174 (16)	−0.0053 (17)
C9	0.0318 (17)	0.0398 (18)	0.052 (2)	0.0035 (15)	0.0049 (15)	0.0017 (17)
C11	0.048 (2)	0.0445 (19)	0.0297 (18)	−0.0072 (17)	−0.0026 (15)	0.0058 (16)
C12	0.060 (2)	0.069 (2)	0.034 (2)	−0.004 (2)	−0.0011 (17)	−0.0014 (19)
C13	0.062 (3)	0.084 (3)	0.049 (3)	0.009 (2)	0.006 (2)	−0.003 (2)
C14	0.097 (4)	0.059 (2)	0.037 (2)	0.002 (3)	0.009 (2)	−0.003 (2)
F14	0.141 (2)	0.112 (2)	0.0519 (16)	0.0258 (17)	0.0209 (15)	−0.0175 (15)
C15	0.086 (3)	0.074 (3)	0.036 (2)	−0.015 (3)	−0.007 (2)	−0.004 (2)
C16	0.053 (2)	0.075 (3)	0.043 (2)	−0.011 (2)	−0.0043 (18)	0.006 (2)

### Geometric parameters (Å, °)

**Table:**

C1—O1	1.210 (4)	C71—H71B	0.9600
C1—C11	1.483 (5)	C71—H71C	0.9600
C1—C2	1.484 (5)	C8—C9	1.372 (5)
C2—C3	1.328 (4)	C8—H8	0.9300
C2—H2	0.9300	C9—H9	0.9300
C3—C4	1.449 (4)	C11—C16	1.385 (5)
C3—H3	0.9300	C11—C12	1.387 (4)
C4—C9	1.391 (4)	C12—C13	1.377 (5)
C4—C5	1.393 (4)	C12—H12	0.9300
C5—C6	1.367 (4)	C13—C14	1.358 (5)
C5—H5	0.9300	C13—H13	0.9300
C6—C7	1.402 (4)	C14—F14	1.357 (4)
C6—Br6	1.890 (3)	C14—C15	1.361 (5)
C7—O7	1.340 (4)	C15—C16	1.372 (5)
C7—C8	1.383 (4)	C15—H15	0.9300
O7—C71	1.440 (4)	C16—H16	0.9300
C71—H71A	0.9600		
			
O1—C1—C11	121.2 (3)	H71A—C71—H71C	109.5
O1—C1—C2	121.1 (3)	H71B—C71—H71C	109.5
C11—C1—C2	117.7 (3)	C9—C8—C7	120.1 (3)
C3—C2—C1	122.0 (3)	C9—C8—H8	119.9
C3—C2—H2	119.0	C7—C8—H8	119.9
C1—C2—H2	119.0	C8—C9—C4	122.0 (3)
C2—C3—C4	128.3 (3)	C8—C9—H9	119.0
C2—C3—H3	115.8	C4—C9—H9	119.0
C4—C3—H3	115.8	C16—C11—C12	118.0 (3)
C9—C4—C5	118.0 (3)	C16—C11—C1	118.9 (3)
C9—C4—C3	119.2 (3)	C12—C11—C1	123.1 (3)
C5—C4—C3	122.8 (3)	C13—C12—C11	121.0 (3)
C6—C5—C4	119.9 (3)	C13—C12—H12	119.5
C6—C5—H5	120.0	C11—C12—H12	119.5
C4—C5—H5	120.0	C14—C13—C12	118.5 (4)
C5—C6—C7	122.0 (3)	C14—C13—H13	120.8
C5—C6—Br6	119.1 (2)	C12—C13—H13	120.8
C7—C6—Br6	118.9 (2)	F14—C14—C13	118.8 (4)
O7—C7—C8	125.5 (3)	F14—C14—C15	118.4 (4)
O7—C7—C6	116.6 (3)	C13—C14—C15	122.8 (4)
C8—C7—C6	117.9 (3)	C14—C15—C16	118.1 (4)
C7—O7—C71	117.0 (3)	C14—C15—H15	120.9
O7—C71—H71A	109.5	C16—C15—H15	120.9
O7—C71—H71B	109.5	C15—C16—C11	121.6 (4)
H71A—C71—H71B	109.5	C15—C16—H16	119.2
O7—C71—H71C	109.5	C11—C16—H16	119.2
			
O1—C1—C2—C3	−8.4 (5)	C7—C8—C9—C4	3.2 (5)
C11—C1—C2—C3	172.8 (3)	C5—C4—C9—C8	−2.0 (4)
C1—C2—C3—C4	177.2 (3)	C3—C4—C9—C8	177.6 (3)
C2—C3—C4—C9	−172.8 (3)	O1—C1—C11—C16	−19.2 (5)
C2—C3—C4—C5	6.7 (5)	C2—C1—C11—C16	159.6 (3)
C9—C4—C5—C6	−0.2 (4)	O1—C1—C11—C12	159.3 (3)
C3—C4—C5—C6	−179.7 (3)	C2—C1—C11—C12	−21.8 (5)
C4—C5—C6—C7	1.2 (4)	C16—C11—C12—C13	−0.6 (5)
C4—C5—C6—Br6	−177.6 (2)	C1—C11—C12—C13	−179.2 (3)
C5—C6—C7—O7	178.8 (3)	C11—C12—C13—C14	0.1 (6)
Br6—C6—C7—O7	−2.3 (4)	C12—C13—C14—F14	−179.2 (3)
C5—C6—C7—C8	−0.2 (5)	C12—C13—C14—C15	0.2 (6)
Br6—C6—C7—C8	178.7 (2)	F14—C14—C15—C16	179.4 (4)
C8—C7—O7—C71	−3.1 (5)	C13—C14—C15—C16	0.1 (6)
C6—C7—O7—C71	178.0 (3)	C14—C15—C16—C11	−0.6 (6)
O7—C7—C8—C9	179.2 (3)	C12—C11—C16—C15	0.9 (6)
C6—C7—C8—C9	−2.0 (5)	C1—C11—C16—C15	179.5 (3)
